# Effects of isolated central nervous system involvement evaluated by multiparameter flow cytometry prior to allografting on outcomes of patients with acute lymphoblastic leukemia

**DOI:** 10.3389/fonc.2023.1166990

**Published:** 2023-05-10

**Authors:** Ling Ma, Lan-Ping Xu, Yu Wang, Xiao-Hui Zhang, Huan Chen, Yu-Hong Chen, Feng-Rong Wang, Wei Han, Yu-Qian Sun, Chen-Hua Yan, Meng Lv, Fei-Fei Tang, Xiao-Dong Mo, Zhi-Dong Wang, Qian Jiang, Jin Lu, Hao Jiang, Yan-Rong Liu, Kai-Yan Liu, Ying-Jun Chang, Xiao-Jun Huang

**Affiliations:** Peking University People’s Hospital, Peking University Institute of Hematology, National Clinical Research Center for Hematologic Disease, Beijing Key Laboratory of Hematopoietic Stem Cell Transplantation, Beijing, China

**Keywords:** acute lymphoblastic leukemia, central nervous system involvement, isolated flow cytometry positive, allogeneic hematopoietic stem cell transplantation, transplant outcomes

## Abstract

**Introduction:**

Allogeneic hematopoietic stem cell transplantation (allo-HSCT) remains a major strategy to cure patients with acute lymphoblastic leukemia (ALL). The aim of this study was to evaluate whether isolated flow cytometry (FCM)-positive central nervous system (CNS) involvement before allo-HSCT is clinically significant.

**Methods:**

The effects of isolated FCM-positive CNS involvement prior to transplantation on the outcomes of 1406 ALL patients with complete remission (CR) were retrospectively investigated.

**Results:**

Patients were classified into isolated FCM-positive CNS involvement (n=31), cytology-positive CNS involvement (n = 43), and negative CNS involvement (n = 1332) groups. Among the three groups, the 5-year cumulative incidence of relapse (CIR) values were 42.3%, 48.8%, and 23.4%, respectively (*P*<0.001). The 5-year leukemia-free survival (LFS) values were 44.7%, 34.9%, and 60.8%, respectively (*P*<0.001). Compared with the negative CNS group (n=1332), the 5-year CIR of the pre-HSCT CNS involvement group (n=74) was higher (46.3% *vs*. 23.4%, *P*<0.001], and the 5-year LFS was inferior (39.1% *vs*. 60.8%, *P*<0.001). Multivariate analysis indicated that four variables, T-cell ALL, in second complete remission or beyond (CR2+) at HSCT, pre-HSCT measurable residual disease positivity, and pre-HSCT CNS involvement, were independently associated with a higher CIR and inferior LFS. A new scoring system was developed using the following four variables: low-risk, intermediate-risk, high-risk, and extremely high-risk groups. The 5-year CIR values were 16.9%, 27.8%, 50.9%, and 66.7%, respectively (*P*<0.001), while the 5-year LFS values were 67.6%, 56.9%, 31.0%, and 13.3%, respectively (*P*<0.001).

**Conclusion:**

Our results suggest that ALL patients with isolated FCM-positive CNS involvement are at a higher risk of recurrence after transplantation. Patients with pre-HSCT CNS involvement had higher CIR and inferior survival outcomes.

## 1 Introduction

Presently, allogeneic hematopoietic stem cell transplantation (allo-HSCT) remains one of the main ways to cure patients with acute lymphoblastic leukemia (ALL) (1–6). However, recurrence after transplantation remains one of the main factors affecting the survival of ALL patients (4, 7). Several risk factors before HSCT, such as complete remission (CR) status (8), positive measurable residual disease (MRD) (9–17), and central nervous system (CNS) involvement (18–20), were associated with relapse in patients with ALL who underwent allo-HSCT. Shigematsu et al. (21) confirmed that ALL patients with CNS involvement who received allografting experienced a higher cumulative incidence of relapse (CIR) and inferior survival compared to those without. Aldoss et al. (22) showed that compared to allografts without CNS involvement pre-HSCT, patients with CNS involvement had a higher risk of CNS relapse (2-year CNS relapse: 9.6% *vs*. 1.4%, *P*<0.0001), inferior event-free survival (EFS) (hazard ratio [HR]: 1.52; *P*=0.003), and worse overall survival (OS, HR: 1.55; *P*=0.003) after transplantation. In allo-HSCT settings, Kharfan-Dabaja et al. (23) demonstrated that CNS involvement at diagnosis was also associated with a significantly higher incidence of relapse (HR: 1.58, *P*=0.03) and a trend towards worse leukemia-free survival (LFS, HR: 1.38, *P*=0.057). However, these studies failed to answer the question of whether isolated flow cytometry (FCM)-positive CNS involvement before transplantation is clinically significant, although available studies (24–28) suggest that FCM is superior to conventional cytology (CC) in identifying leukemia cells in cerebrospinal fluid (CSF).

In our previous study (29), we found that transplant patients with ALL could be classified into subgroups with high and low risk of relapse according to pre-HSCT disease status, immunophenotype of ALL, and MRD before transplantation. However, the effects of isolated FCM-positive CNS involvement before transplantation on outcomes in these subgroup patients is unclear because of the low number of participants enrolled in the studies reported by other researchers (18, 22). In addition, available studies performed by others (9, 30) and our previous studies (17, 29, 31) suggest that the risk score system is superior to single variables in predicting transplant outcomes. However, data are lacking on whether incorporating positive CNS involvement with other variables, including disease status, immunophenotype of ALL, and MRD status, before transplantation could further improve the risk stratification of patients with ALL. Therefore, we performed a retrospective study to investigate the association of isolated FCM positivity in CNS before transplantation with outcomes in all patients with ALL and subgroup cases who underwent allo-HSCT. We also established a risk score system based on positive CNS involvement and other variables to improve the stratification of transplant outcomes.

## 2 Patients and methods

### 2.1 Study design

This retrospective analysis included 1406 patients with ALL who underwent allo-stem cell transplantation at Peking University People’s Hospital between January 2009 and December 2018. All participants signed an informed consent document and had relatively complete medical records. The study protocol was in accordance with the Declaration of Helsinki and was approved by the Institutional Review Board of Peking University. All patients were treated according to the transplantation protocol, as previously described (32).

### 2.2 Transplantation protocols

Granulocyte colony-stimulating factor (G-CSF, 5 μg/kg/d for 5 days) was used to mobilize the bone marrow (G-BM) or peripheral blood (G-PB). The target mononuclear cell count in the total allografts was greater than 6×10^8^ cells/kg of recipient weight.

For patients who received haploidentical transplantation, the Bu-based conditioning regimen was as follows: cytarabine (4 g/m^2^/d) on days –10 to –9, busulfan (3.2 mg/kg/d) on days –8 to –6, cyclophosphamide (CTX, 1.8 g/m^2^/d) on days –5 to –4, oral Me-CCNU (250 mg/m^2^, once) on day –3, and anti-thymocyte globulin (ATG, 2.5 mg/kg/d) on days –5 to –2. The total body irradiation (TBI)-based conditioning regimen consisted of TBI (770 cGy) on day –6, CTX (1.8 g/m^2^/d) on days –5 to –4, oral Me-CCNU (250 mg/m^2^, once) on day –3, and ATG (2.5 mg/kg/d) on days –5 to –2. Patients who underwent human leukocyte antigen-matched sibling donor transplantation received the same regimen mentioned above, but without ATG. The graft-versus-host disease (GVHD) prophylaxis regimen comprised immunosuppressive agents, including cyclosporine A, mycophenolate mofetil, and short-term methotrexate. The detailed protocol is described in our previous publication (32).

### 2.3 Investigation of CNS involvement

Fresh CSF samples were collected, centrifuged, and stained for morphological examination. An expert cytopathologist interpreted each case. Positive cytology was defined as unequivocal morphological evidence of leukemic blasts in the CSF.

Simultaneously, CSF samples were examined by multiparameter FCM, which was performed using a combination of six-to-eight color antibodies, according to the immunophenotype of blasts identified in the bone marrow at the initial diagnosis. The antibody combination panel consisted of mCD3, CD2, CD4, CD5, CD7, CD8, CD10, CD19, CD20, CD34, CD38, CD45, CD58, and CD123. FCM positivity was considered when a cluster of more than 10 cells was characterized by a leukemia-associated immunophenotype (33).

### 2.4 Definitions

Hyperleukocytosis was defined as leukocyte count greater than 30×10^9^/L in B cell ALL (B-ALL) or 100×10^9^/L in T cell ALL (T-ALL). Patients were classified as high-risk if they met the following criteria: age >35 years, hyperleukocytosis, adverse cytogenetics (t[4;11], complex karyotype, low hypodiploidy-near triploidy), or delayed CR1 (remission required more than 4 weeks after induction of therapy) (34). CNS involvement was defined as infiltration of leukemic blasts in the CSF, as determined by either FCM, CC, or both methods. Isolated FCM-positive CNS involvement was defined as infiltration in the CNS detected only by multiparameter FCM (33). Hematological relapse was diagnosed when blasts reappeared in the PB or >5% in the BM aspirate. Extramedullary recurrence was diagnosed through physical examination, imaging, pathology, or cytology. The cumulative incidence rate of hematological recurrence (CIHR) involves only hematological recurrence, whereas the CIR involves hematological recurrence and extramedullary recurrence. OS was defined from the day of transplantation to the day of death for any reason or to the last day of follow-up. Leukemia-free survival (LFS) was defined from the date of transplantation as the starting point to the date of the first event or last follow-up as the endpoint. The events for LFS included morphological or extramedullary relapse and death from any cause. Non-relapse mortality (NRM) was defined as death from any cause within 28 days after HSCT or death without evidence of disease recurrence after 28 days. Neutrophil recovery was defined as the first day of an absolute neutrophil count that exceeded 0.5×10^9^/L for 3 consecutive days. Platelet engraftment was defined as the first of 7 consecutive days with a platelet count exceeding 20×10^9^/L without platelet transfusion support. Acute and chronic GVHD was defined as previously described (35).

The endpoint of the last follow-up for all surviving patients was June 18, 2022.

### 2.5 Statistical analysis

Patient characteristics were compared among the positive blasts in CSF detected by CC or FCM and negative subgroups using the Chi-square test for categorical variables and Mann–Whitney test for continuous variables. Cumulative incidence curves were used with a competing risk setting, and relapse was treated as a competing event to calculate the NRM probability. Death from any cause was a competing risk for relapse. The probabilities of LFS, OS, incidence of hematological relapse, and NRM were estimated using the Kaplan–Meier method and log–rank test. Age, sex, immunophenotype of ALL, hyperleukocytosis at diagnosis, risk stratification, BCR/ABL positive or negative at diagnosis, and concomitant extramedullary (except CNS involvement) or not at diagnosis were included in the univariate analysis for CNS involvement. Pre-HSCT isolated FCM-positive CNS involvement, cytology-positive or negative CNS involvement, and all variables in **Table 1**, except for donor-recipient sex-matched graft, were included in the univariate analysis for the outcomes. Parameters with *P ≤* 0.1 were selected to enter the multivariable analysis using the Cox proportional hazards model. In the multivariate model, the proportional hazard assumption and linear relation between covariates were verified. *P ≤* 0.05 indicated a significant difference. SPSS version 26.0 software (IBM Corporation, Armonk, NY, USA) and RStudio were used to perform the statistical analysis.

**Table 1:** FCM and CC analysis of CSF for the detection of CNS involvement in patients with ALL (N=1406).

Conventional cytology	Flow cytometry			P value*
	positive	Negative	Total	
Positive	43	0	43	<0.001
negative	31	1332	1363	
Total	74	1332	1406

FCM, Flow cytometry; CC, Conventional cytology; *Chi-square t.

## 3 Results

### 3.1 Patient characteristics and transplant outcomes

From January 2009 to December 2018, 1406 ALL patients with CR underwent allo-HSCT at our center, and before transplantation, blasts were detected in the CSF of 74 patients (5.4%). Forty-three patients (3.1%) had cytology-positive CNS involvement, which was detected by both FCM and CC, and 31 patients (2.2%) had isolated FCM-positive CNS involvement (**Table 1**). Twenty-two patients (51.2%) and 14 patients (45.2%) in the cytology-positive and isolated FCM-positive groups, respectively, were detected at diagnosis. The remaining patients were detected during treatment, and none of them was detected at transplantation after treatment with intrathecal injection. The baseline characteristics of the isolated FCM-positive, cytology-positive, and negative CNS involvement groups are presented in **Table 2**. The age of the patients with CNS involvement was lower (*P*=0.001). The incidence rates of hyperleukocytosis at diagnosis (*P*=0.024) and BCR/ABL positive (*P*=0.046) were higher in the cytology-positive group. Patients with extramedullary involvement (except CNS involvement) at diagnosis (*P*<0.001) and in second complete remission or beyond (CR2+) at transplantation were more common in the isolated FCM-positive or cytology-positive group (*P*<0.001).

**Table 2:** Baseline characteristics of ALL patients with or without CNS involvement prior to HSCT.

Characteristics	Isolated FCM-positive group N=31	Cytology-positive group N=43	Negative CNS group N=1332	*P* value
Median age (range), years	19 (2–33)	21 (2–59)	26(1-64)	0.001
Male, n (%)	23(74.2)	30(69.8)	791(59.4)	0.104
Diagnosis, n (%)				0.462
B-ALL	22(71.0)	35(81.4)	1063(79.8)	
T-ALL	9(29.0)	8(18.6)	269(20.2)	
Hyperleukocytosis at diagnosis, n (%)	7(22.6)	22(52.4)	460(35.5)	0.024
BCR/ABL positive at diagnosis, n (%)	3(9.7)	14(32.6)	399(30.0)	0.046
Risk stratification, n (%)				0.208
Standard-risk group	18(58.1)	17(39.5)	568(42.6)	
High-risk group	13(41.9)	26(60.5)	764(57.4)	
Concomitant extramedullary (except CNS involvement) at diagnosis, n (%)	5(16.1)	6(14.0)	99(7.4)	0.021
Grafts, n (%)				0.468
PB	0	2(4.7)	37(2.8)	
BM+PB	31(100)	41(95.3)	1295(97.2)	
Transplant modality, n (%)				0.406
Haplo-SCT	27(87.1)	32(74.4)	1064(79.9)	
MSDT	4(12.9)	11(25.6)	268(20.1)	
Disease status at HSCT, n (%)				<0.001
CR1	21(67.7)	29(67.4)	1159(87.0)	
CR2+	10(32.3)	14(32.6)	173(13.0)	
Pre-MRD positive, n (%)	7(22.6)	9(20.9)	291(21.8)	0.985
ABO matched graft, n (%)	19(61.3)	24(55.8)	753(56.5)	0.865
Donor-recipient sex-matched graft, n (%)				0.458
Male-male	17(54.8)	17(39.5)	530(39.8)	
Male-female	6(19.4)	8(18.6)	355(26.7)	
Female-male	6(19.4)	12(27.9)	268(20.1)	
Female-female	2(6.5)	6(14.0)	179(13.4)	
Conditioning regimen, n (%)				<0.001
BU-based	26(83.9)	42(97.7)	1298(97.4)	
TBI-based	5(16.1)	1(2.3)	34(2.6)	

HSCT, hematopoietic stem cell transplantation; ALL, acute lymphoblastic leukemia; CNS, central nervous system; isolated FCM-positive, the blast cells in the CSF was detected only by Flow cytometry; cytology-positive, the blast cells in the CSF was detected by conventional cytology; CNS involvement, blasts from CSF were detected; negative CNS, blasts from CSF were not detected; PB, peripheral blood; BM, bone marrow; CR, complete remission; haplo-SCT, haploidentical stem cell transplantation; MSDT, matched sibling donor transplantation; CR2+, second complete remission or beyond; Pre-MRD, pre-transplantation measurable residual disease; MRD , measurable residual disease; BU, busulfan; TBI, total body irradiation.

The results are presented in **Table 3**. Of the 74 patients with pre-HSCT CNS involvement, 18 (24.3%) had CNS involvement recurrence after transplantation (post-HSCT CNS involvement). Among the 31 patients in the isolated FCM-positive CNS involvement group before transplantation, six patients were confirmed to have isolated FCM-positive CNS involvement and two patients had cytology-positive CNS involvement after transplantation. Of the 43 patients in the cytology-positive CNS involvement group before transplantation, nine patients had cytology-positive CNS involvement after transplantation. Of the remaining 1332 patients with negative CNS involvement before transplantation, 52 (3.9%) had post-HSCT CNS involvement, including 22 with isolated FCM-positive CNS involvement and 30 with cytology-positive CNS involvement. Among the 1406 patients with allograft ALL with a median follow-up of 56.5 months, the estimated 5-year OS rate was 65.0% (95% confidence interval [CI]: 62.5–67.6%), the 5-year LFS rate was 59.8% (95% CI: 57.3–62.3%), the 5-year CIHR was 23.5% (95% CI: 21.3–25.7%), the 5-year CIR was 25.1% (95% CI: 22.8–27.4%), and the 5-year NRM rate was 15.3% (95% CI: 13.4–17.2%).

**Table 3:** Outcomes of patients with or without CNS involvement following allo-HSCT for ALL.

outcomes	Isolated FCM-positive N=31	Cytology-positive N=43	Negative CNS group N=1332	*P* value
Engraftment (range), days				
Neutrophil engraftment	13(10-25)	14(9-21)	13(9-66)	0.973
Platelet engraftment	14(9-693)	15(5-124)	15(4-418)	0.119
Acute GVHD (grades), n (%)				
I-II	18(58.1)	11(26.2)	542(42.1)	0.223
III-IV	1(3.6)	1(3.4)	45(4.5)	0.939
Chronic GVHD, n (%)	11(35.5)	12(27.9)	579(43.5)	0.124
Post-HSCT CNS involvement, n (%)				
Isolated FCM-positive	6(19.4)	0	22(1.7)	<0.001
Cytology-positive	2(6.5)	9(20.9)	30(2.3)	<0.001
5 years of CIHR (95%*CI*)	42.3 (24.9-59.7)	44.2 (29.3-59.0)	22.3 (20.1-24.5)	<0.001
5 years of CIR (95%*CI*)	42.3 (24.9-59.7)	48.8 (33.9-63.8)	23.4 (21.1-25.6)	<0.001
5 years of NRM (95%*CI*)	9.7 (0-20.1)	16.3 (5.2-27.3)	15.3 (13.4-17.3)	0.506
5 years of LFS (95%*CI*)	44.7 (27.1-62.3)	34.9 (20.6-49.2)	60.8 (58.3-63.3)	<0.001
5 years of OS (95%*CI*)	54.6 (37.0-72.2)	44.2 (29.3-59.0)	66.0 (63.5-68.5)	0.002

HSCT, hematopoietic stem cell transplantation; ALL, acute lymphoblastic leukemia; CNS, central nervous system; isolated FCM-positive, the blast cells in the CSF was detected only by Flow cytometry; cytology-positive, the blast cells in the CSF was detected by conventional cytology; CNS involvement, blasts from CSF were detected; negative CNS, blasts from CSF were not detected; GVHD, graft-versus-host disease; Post-HSCT CNS involvement, post-transplantation blasts from CSF were detected; CIHR, the cumulative incidence rate of hematological recurrence; CIR, the cumulative incidence rate of relapse including hematological and extramedullary; NRM, non-relapse mortality; OS, overall survival; LFS, leukemia-free survival.

### 3.2 Risk factors for pre-HSCT CNS involvement

Univariate analysis showed that age above 25 years (median) (*P*<0.001), male patients (*P*=0.039), and concomitant extramedullary at diagnosis (*P*=0.024) were associated with CNS involvement before transplantation. Further multivariate analysis showed that only age above 25 years (median) (odds ratio: 0.395; 95% CI: 0.228–0.632; *P*<0.001) was an independent prognostic factor for pre-HSCT CNS involvement (**Table S1**).

### 3.3 Association of isolated FCM-positive CNS involvement pre-HSCT with transplant outcomes

**Figure 1:**
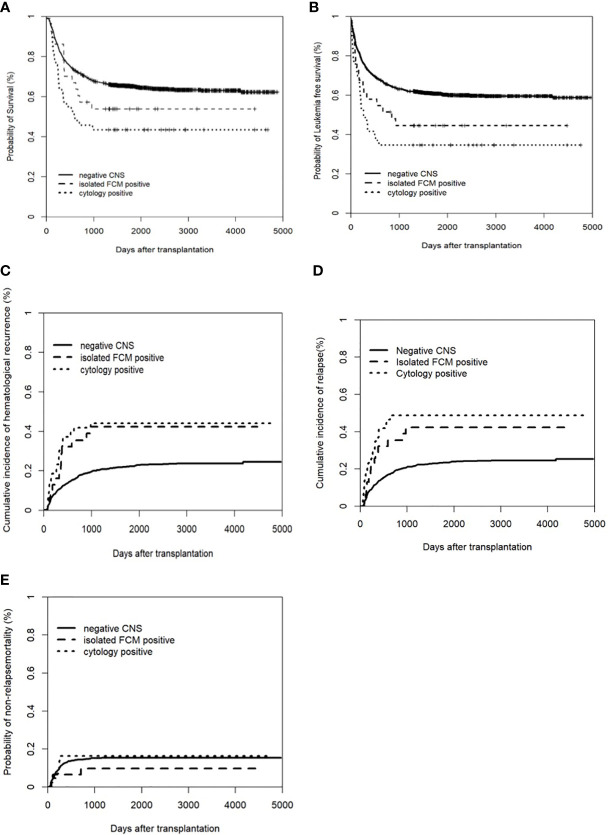
Clinical outcomes of patients with and without CNS involvement. **(A)** Overall survival (OS); **(B)** Leukemia free of survival (LFS): * Leukemia free survival was defined as survival without hematologic relapse or extramedullary relapse; **(C)** cumulative incidence of hematological recurrence; **(D)** cumulative incidence of relapse; **(E)** non-relapse mortality (NRM).

Among the isolated FCM-positive CNS involvement group, the cytology-positive CNS involvement group, and negative CNS group, the 5-year CIHR was 42.3% (95% CI: 24.9–59.7%), 44.2% (95% CI: 29.3–59.0%), and 22.3% (95% CI: 20.1–24.5%), respectively (*P*<0.001). The 5-year CIR was 42.3% (95% CI: 24.9–59.7%), 48.8% (95% CI: 33.9–63.8%), and 23.4% (95% CI: 21.1–25.6%), respectively (*P*<0.001). Compared to the negative CNS group, the isolated FCM-positive CNS involvement group had a higher CIHR (*P*=0.007) and CIR (*P*=0.003), and the cytology-positive CNS involvement group had a higher CIHR (*P*<0.001) and CIR (*P*<0.001). There were no significant differences in the CIHR (*P*=0.761) or CIR (*P*=0.662) between the two pre-HSCT CNS involvement groups (**Figure 1**). We assessed the risk factors that might influence outcomes in ALL patients undergoing allo-HSCT. The univariate analysis results are shown in **Table S2**. On multivariate analysis, T-ALL, CR2+ at HSCT, pre-HSCT MRD positivity, and pre-HSCT cytology-positive CNS involvement were independent risk factors associated with higher CIHR, CIR, and inferior OS and LFS. Pre-HSCT isolated FCM-positive CNS involvement was the only independent risk factor associated with higher CIHR and CIR. Details of the multivariate analyses of the outcomes are shown in **Table 3**. Univariate and multivariate analyses showed that acute GVHD grades III–IV were the only independent risk factors for NRM (data not shown).

### 3.4 Association of pre-HSCT CNS involvement with transplant outcomes

Considering the higher CIR of patients either in the isolated FCM-positive CNS involvement group or in the cytology-positive CNS involvement group compared to those in the negative CNS group, we investigated the association of pre-HSCT CNS involvement determined either by FCM or cytology. The 5-year CIHR of patients with pre-HSCT CNS involvement was 43.4% (95% CI: 32.1–54.7%), which was significantly higher than that of the negative CNS (22.3%, 95% CI: 20.1–24.5%, *P*<0.001), and the 5-year CIR was 46.3% (95% CI: 34.7–57.4%) *vs*. 23.4% (95% CI: 21.1–25.6%) (*P*<0.001). The 5-year LFS rate of patients with pre-HSCT CNS involvement was lower (39.1% [95% CI: 27.9–50.3%] *vs*. 60.8% [95% CI: 58.3–63.3%], *P*<0.001), and the 5-year OS rate of pre-HSCT CNS involvement was also inferior (48.6% [95% CI: 37.2–60.0%] *vs*. 66.0% [95% CI: 63.5–68.5%], *P*=0.001). There was no significant difference in NRM between the two groups (13.5% [95% CI: 5.7–21.3%] *vs*. 15.3% [95% CI: 13.4–17.3%], *P*=0.679). We then assessed the risk factors that might influence the outcomes; the univariate analysis results are shown in **Table S2**. On multivariate analysis, T-ALL, CR2+ at HSCT, pre-HSCT MRD positivity, and pre-HSCT CNS involvement were independent risk factors associated with higher CIHR and CIR and inferior OS and LFS. Details of the multivariate analyses of the outcomes are shown in **Table 4**. Univariate and multivariate analyses showed that acute GVHD grades III–IV were the only independent risk factors for NRM.

**Table 4:** Multivariate analysis of the factors associated with CIHR, CIR LFS and OS about isolated FCM-positive CNS involvement group.

Covariates*	CIHR		CIR		LFS		OS	
	HR (95%*CI*)	*P* value	HR (95%*CI*)	*P* value	HR (95%*CI*)	*P* value	HR (95%*CI*)	*P* value
Diagnosis (T-ALL vs B-ALL)	1.420 (1.063-1.900)	0.018	1.470 (1.102-1.960)	0.009	1.330 (1.076-1.644)	0.008	1.500 (1.204-1.869)	<0.001
BCR/ABL positive at diagnosis	0.853 (0.658-1.100)	0.230	0.949 (0.740-1.220)	0.680				
Concomitant extramedullary (except CNS involvement) at diagnosis	1.221 (0.821-1.820)	0.320	1.262 (0.858-1.860)	0.240	1.328 (0.992-1.777)	0.057	1.334 (0.987-1.803)	0.061
Disease status at HSCT (CR2+ vs CR1)	2.085 (1.592-2.730)	<0.001	2.002 (1.530-2.620)	<0.001	1.944 (1.575-2.400)	<0.001	1.923 (1.537-2.407)	<0.001
Pre-HSCT MRD positive	1.970 (1.558-2.490)	<0.001	2.051 (1.635-2.570)	<0.001	1.588 (1.321-1.909)	<0.001	1.461 (1.197-1.783)	<0.001
Pre-HSCT CNS								
Negative CNS	1		1		1		1	
Isolated FCM-positive	1.832 (1.070-3.140)	0.027	1.765 (1.036-3.01)	0.037	1.333 (0.820-2.167)	0.246	1.160 (0.679-1.981)	0.586
Cytology-positive	2.245 (1.372-3.670)	0.001	2.524 (1.556-4.100)	<0.001	1.985 (1.351-2.915)	<0.001	1.819 (1.202-2.752)	0.005

CIHR, the cumulative incidence rate of hematological recurrence; CIR, the cumulative incidence rate of relapse including hematological and extramedullary; LFS, leukemia-free survival; OS, overall survival; CR, complete remission; CR2+, second complete remission or beyond; Pre-HSCT MRD, pre-transplantation measurable residual disease; MRD, measurable residual disease; Pre-HSCT CNS involvement, pre-transplantation blasts from CSF were detected; isolated FCM-positive, the blast cells in the CSF was detected only by Flow cytometry; cytology-positive, the blast cells in the CSF was detected by conventional cytology; negative CNS, blasts from CSF were not detected; HR, hazard ratio; CI, confidence interval.

* All variables were first included in the univariate analysis; only variables with P< 0.1 were included in the Cox proportional hazards model with time-dependent variables.

### 3.5 A new scoring system with transplant outcomes in the entire cohort of patients

**Figure 2:**
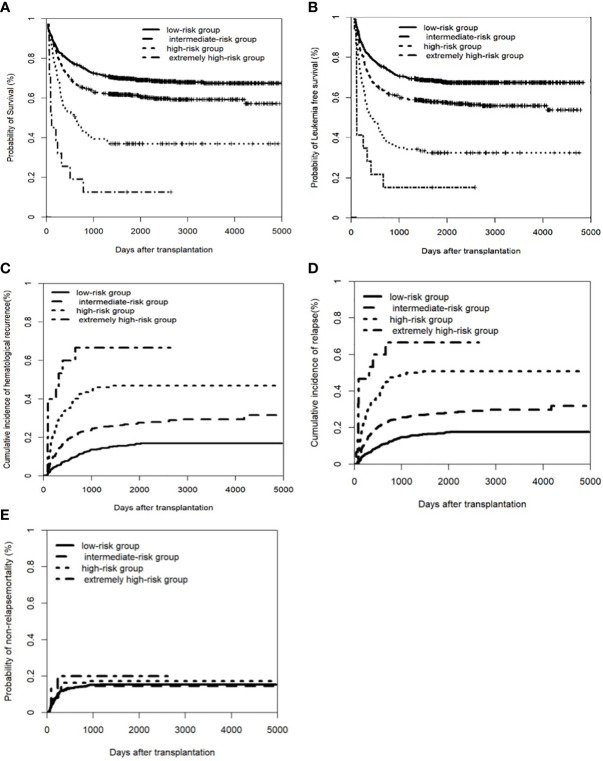
Clinical outcomes of a new scoring system in the entire cohort of patients. The factors including pre-CNS involvement, pre-HSCT MRD postive, disease status of CR2+ before transplant, and T-ALL. **(A)** Overall survival (OS); **(B)** Leukemia free of survival (LFS): * Leukemia free survival was defined as survival without hematologic relapse or extramedullary relapse; **(C)** cumulative incidence of hematological recurrence; **(D)** cumulative incidence of relapse; **(E)** non-relapse mortality (NRM).

Based on the multivariate analysis (**Table S1**), we developed a risk score for transplant outcome prediction. The score was the number of risk factors, including T-ALL, CR2+ at HSCT, pre-HSCT MRD positivity, and pre-HSCT CNS involvement. By combining the risk scores for these four major variables, patients were stratified into four distinctive risk groups: low-risk, intermediate-risk, high-risk, and extremely high-risk, which had scores of 0, 1, 2, and 3–4, respectively. The 5-year CIR of the four groups was 16.9% (95% CI: 14.1–19.7%), 27.8% (95% CI: 24.0–31.6%), 50.9% (95% CI: 42.0–59.8%), and 66.7% (95% CI: 40.3–93.1%), respectively; the 5-year LFS was 67.6% (95% CI: 64.3–70.9%), 56.9% (95% CI: 52.3–61.0%), 31.0% (95% CI: 22.8–39.2%), and 13.3% (95% CI: 0–30.5%), respectively; and the 5-year OS was 71.8% (95% CI: 68.5–75.1%), 63.4% (95% CI: 59.3–67.5%), 38.3% (95% CI: 29.7–46.9%), and 13.3% (95% CI: 0–30.5%), respectively (all *P*<0.001) (**Figure 2**). The multivariate analysis indicated that the new scoring system was the only independent risk factor simultaneously associated with higher CIHR and CIR and inferior OS and LFS. In addition, concomitant extramedullary involvement at diagnosis was another factor that affected OS (**Table S2**).

### 3.6 Subgroup analysis of pre-HSCT CNS involvement compared to negative CNS

As shown in **Table S3**, in the B-ALL or T-ALL subgroup, disease status of the CR1 stage subgroup or CR2+ subgroup, and pre-HSCT MRD negative subgroup or pre-HSCT MRD positive subgroup, patients with CNS involvement pre-HSCT had significantly higher CIHR or CIR than those with negative CNS pre-HSCT. Regarding survival, the cases of CNS involvement pre-HSCT from all the other subgroups, except those from the CR2+ subgroup, had worse LFS and OS. In addition, cases with CNS involvement pre-HSCT in the T-ALL subgroup, disease status of CR1 stage subgroup, pre-HSCT MRD negative subgroup, and pre-HSCT MRD positive subgroup had an inferior OS compared to those with negative CNS. The multivariate analysis indicated that pre-HSCT CNS involvement was an independent risk factor associated with higher CIHR and CIR and inferior OS and LFS in each subgroup (data not shown).

The participants (n=1406) were divided into three groups according to age at diagnosis: 199 cases (14.2%), pediatric (1~14 years); 944 cases (67.2%), adolescent and young adult (AYA) (15~39 years); and 263 cases (18.7%), adults (age above 39 years), respectively. Among the three groups, no statistical differences were observed in OS (*P*= 0.154), LFS (*P*=0.135), and relapse (*P*=0.660). Subgroup analysis showed that CNS involvement in pediatric or adult patients was not independently associated with OS, LFS, relapse, and NRM. In the AYA group, isolated FCM-positive CNS involvement before transplantation had a higher risk of recurrence after transplantation and worse LFS and OS compared to negative CNS; these results were similar to those of patients with cytology-positive CNS involvement (data not shown).

Among the above subgroups, there was no difference in NRM.

## 4 Discussion

To our knowledge, this is the first study to show that isolated FCM-positive CNS involvement pre-HSCT was associated with higher CIR after transplantation (**Tables 2**–**4**). The negative effects of pre-HSCT CNS involvement by either cytology or FCM on transplant outcomes of ALL patients were demonstrated in both the total patient group and subgroups of total patients (**Tables S1**, **S3**). More importantly, a new scoring system based on four risk factors, including pre-HSCT CNS involvement, could better predict transplant outcomes (**Figure 2**; **Table S2**). Our study suggests that pre-HSCT CNS involvement by either cytology or FCM is an independent variable for predicting transplant outcomes in patients with ALL.

Regarding the clinical significance of CNS involvement detected by FCM, previous studies have demonstrated that CNS involvement by FCM at diagnosis is associated with a higher risk of relapse in childhood ALL (36, 37). In adult ALL/lymphoblastic lymphoma patients, Del Principe et al. (38) analyzed the association of CNS involvement in 38 newly diagnosed patients; 53% of the patients received allo-HSCT. The results showed that 2-year OS rates were 0%, 22%, and 53% (*P*=0.008) for the FCM+/CC+, FCM+/CC–, and FCM–/CC– subgroup, respectively. Gong et al. (33) found that the OS of patients with FCM+/CC– was similar to that of patients with FCM+/CC+, both of which were significantly shorter than those of patients with FCM–/CC–; however, only 142 patients in the study received allo-HSCT. In another retrospective study, Del Principe et al. (39) analyzed the data from 13 Italian hematological centers and found that patients with CNS involvement detected by FCM or CC had similar hematological recurrence rates, disease-free survival, and OS; however, compared to patients without CNS involvement, they had higher relapse rates and worse survival. In this study, only 55.1% of the patients underwent allo-HSCT. Consistent with studies in childhood ALL (36, 37) and the study by Del Principe et al. (39), we found that isolated FCM-positive CNS involvement before transplantation had a higher risk of recurrence after transplantation, including higher CIHR or CIR, which was similar to those with cytology-positive CNS involvement. In contrast to the studies by Del Principe et al. (38), our study showed that the isolated FCM-positive CNS involvement was not independently associated with a worse OS on multivariate analysis. Several factors may account for the differences in the effects of isolated FCM-positive CNS involvement on survival between the results of other studies (33, 38, 39). 1) The heterogeneity of treatment in the studies by others (33, 38, 39), but only including allo-HSCT in our study; 2) the differences in leukemia burden between isolated FCM-positive and cytology-positive CNS involvement, and 3) the graft-versus-leukemia effects of allo-HSCT might overcome the negative effect of isolated FCM-positive CNS involvement on survival.

Since the CIR of patients with isolated FCM-positive CNS involvement or cytology-positive CNS involvement was higher than that of patients with negative CNS pre-HSCT in our study, we combined the two groups into one group, namely, pre-HSCT CNS involvement. Our data showed that patients with pre-HSCT CNS involvement had a higher CIHR and CIR, as well as inferior survival, compared to those with negative CNS involvement. Previous studies (21, 22, 40, 41) have shown inconsistent results regarding whether CNS involvement has an adverse effect on the survival of ALL patients. For adult patients with ALL who received either chemotherapy alone or allo-HSCT, Lazarus et al. (40) showed that CNS involvement at diagnosis was an independent risk factor for OS (*P*=0.03), but not for EFS (*P*=0.07). Data from 69 patients with CNS involvement showed that allo-HSCT improved the survival of patients with CNS involvement compared with chemotherapy (43% *vs*. 26%). Hamdi et al. (41) showed that pre-transplant CNS involvement was associated with post-transplant CNS recurrence but did not affect survival. Shigematsu et al. (21) studied 2582 patients with ALL who underwent adult allogeneic stem cell therapy and showed that patients with CNS involvement pre-HSCT had a higher CNS recurrence rate (*P* =0.02) or overall recurrence rate (*P*<0.01), and a worse 3-year OS rate (*P*<0.01). Aldoss et al. (22) demonstrated that pre-HSCT CNS involvement was an independent risk factor for EFS and OS. Although controversy remains (21, 22, 41), most of these studies, including our study, have shown the negative effects of CNS involvement pre-HSCT on transplant outcomes, suggesting that CNS involvement pre-HSCT is a risk factor for poor prognosis in patients with ALL.

A previous study indicated that disease status, pre-HSCT MRD, and immunophenotype of leukemia cells were independent risk factors for CIR and survival in ALL patients who received allografting (29). Therefore, we investigated the effects of pre-HSCT CNS involvement on transplant outcomes of patients in each subgroup. In contrast to other studies, we found that pre-HSCT CNS involvement was associated with a higher CIR in the total and subgroups of patients (**Table S3**). Except for patients with CR2+, cases in the total group and other subgroups with pre-HSCT CNS involvement were associated with poor survival (**Table S3**). We speculate that the lack of negative effects of CNS involvement before HSCT on survival may be explained by the small number of patients with CR2+. Therefore, a large sample of ALL patients with pre-HSCT CNS involvement is needed to confirm our hypothesis.

Based on the results of the multivariate analysis (**Table S1**), we established a new prognostic scoring system combining this with risk factors of disease status, immunophenotype of ALL, and pre-HSCT MRD that affected the transplant outcomes of ALL patients in our previous study (29). Thus, patients were stratified into four distinct risk groups. The risk categories were significantly different for relapse, LFS, and OS, thus providing evidence for more individualized and precise clinical treatment.

This study has some limitations. First, it was a retrospective study; therefore, more multicenter prospective studies are required to confirm our findings. Second, the patients included in our study underwent haploidentical allograft transplants and matched sibling donor transplantation; thus, more studies need to be conducted to confirm this in other transplant modalities, such as matched unrelated donor transplants and umbilical cord blood transplant. Third, our study was conducted only in the allograft model based on G-CSF and ATG-induced immune tolerance; whether the same conclusion can be reached in the allograft of the PT/CY mode (42) requires additional studies. In addition, the number of cases of TBI-based conditioning regimen in our study was small. The role of TBI in ALL patients with CNS involvement should be elucidated in further studies.

## 5 Conclusions

In this large sample study, we found that isolated FCM-positive CNS involvement pre-HSCT was associated with a higher relapse rate but had no effect on survival after transplantation. Pre-HSCT CNS involvement affects both relapse and survival rates. Our results not only provide further evidence suggesting that pre-HSCT CNS involvement, determined by cytology or FCM, is a risk factor for poor outcomes, but also provide a new scoring system based on pre-HSCT CNS involvement and other risk factors for a more precise evaluation of clinical prognosis. These two factors might be helpful for individualized therapy for ALL patients receiving allografting.

## Data availability statement

The original contributions presented in the study are included in the article/**Supplementary Material**. Further inquiries can be directed to the corresponding author.

## Author contributions

XJH and YJC designed the study and revised the paper. LM and YJC collected the data, analyzed the data, and drafted the manuscript. All authors contributed to the article and approved the submitted version.
