# Supplementary material for: Effects of isolated central nervous system involvement evaluated by multiparameter flow cytometry prior to allografting on outcomes of patients with acute lymphoblastic leukemia
Source: Front Oncol. 2023 May 10;13:1166990. doi: 10.3389/fonc.2023.1166990 (PMC10209422; doi:10.3389/fonc.2023.1166990)
Supplement: Supplementary file 1 [file Table_1.docx]

**Table S1 Multivariate analysis of the factors associated with CIHR, CIR LFS and OS about pre-HSCT CNS involvement**

| **Covariates** | **CIHR** | | **CIR** | | **LFS** | | **OS** | |
| --- | --- | --- | --- | --- | --- | --- | --- | --- |
|  | HR  (95%*CI*) | *P*  value | HR  (95%*CI*) | *P*  value | HR  (95%*CI*) | *P*  value | HR  (95%*CI*) | *P*  value |
| Diagnosis (T-ALL vs B-ALL) | 1.420  (1.060-1.890) | 0.020 | 1.458  (1.095-1.940) | 0.010 | 1.326  (1.073-1.638) | 0.009 | 1.492  (1.197-1.858) | <0.001 |
| BCR/ABL positive at diagnosis | 1.170  (0.900-1.510) | 0.240 | 0.958  (0.747-1.230) | 0.740 |  |  |  |  |
| Concomitant extramedullary at diagnosis | 1.230  (0.825-1.820) | 0.310 | 1.268  (0.863-1.860) | 0.230 | 1.313  (0.981-1.758) | 0.068 | 1.332  (0.985-1.801) | 0.063 |
| Disease status at HSCT (CR2 and above vs CR1) | 2.090  (1.593-2.730) | <0.001 | 2.004  (1.532-2.620) | <0.001 | 1.951  (1.581-2.407) | <0.001 | 1.921  (1.534-2.405) | <0.001 |
| Pre-HSCT MRD positive | 1.960  (1.555-2.480) | <0.001 | 2.043  (1.629-2.560) | <0.001 | 1.583  (1.317-1.903) | <0.001 | 1.457  (1.194-1.779) | <0.001 |
| Pre-HSCT CNS involvement | 2.050  (1.414-2.990) | <0.001 | 2.165  (1.500-3.130) | <0.001 | 1.676  (1.231-2.282) | 0.001 | 1.504  (1.076-2.104) | 0.017 |

**Abbreviations:** CIHR=the cumulative incidence rate of hematological recurrence; CIR=the cumulative incidence rate of relapse including hematological and extramedullary; LFS=leukemia-free survival; OS=overall survival; CR=complete remission; Pre-HSCT MRD=pre-transplantation measurable residual disease; Pre-HSCT CNS involvement=pre-transplantation blasts from CSF were detected; HR=hazard ratio; CI=confidence interval;

* All variables were first included in the univariate analysis; only variables with *P*< 0.1 were included in the Cox proportional hazards model with time-dependent variables.

**Table S2 Multivariate analysis of the factors associated with CIHR, CIR LFS and OS about the new scoring system**

| **Covariates** | **CIHR** | | **CIR** | | **LFS** | | **OS** | |
| --- | --- | --- | --- | --- | --- | --- | --- | --- |
|  | HR  (95%*CI*) | *P*  value | HR  (95%*CI*) | *P*  value | HR  (95%*CI*) | *P*  value | HR  (95%*CI*) | *P*  value |
| Concomitant extramedullary at diagnosis | 1.117  (0.761-1.640) | 0.570 | 1.160  (0.802-1.690) | 0.420 | 2.242  (0.938-1.644) | 0.130 | 1.352  (1.011-1.808) | 0.042 |
| New risk scoring system |  |  |  |  |  |  |  |  |
| low-risk | 1 |  | 1 |  | 1 |  | 1 |  |
| intermediate-risk | 1.811  (1.412-2.320) | <0.001 | 1.800  (1.409-2.300) | <0.001 | 1.472  (1.230-1.762) | <0.001 | 1.340  (1.101-1.631) | 0.003 |
| high-risk | 3.606  (2.580-5.040) | <0.001 | 3.870  (2.811-5.340) | <0.001 | 2.864  (2.229-3.679) | <0.001 | 2.648  (2.027-3.459) | <0.001 |
| extremely high-risk | 7.417  (3.459-15.900) | <0.001 | 7.450  (3.382-16.42) | <0.001 | 6.354  (3.630-11.122) | <0.001 | 6.379  (3.612-11.264) | <0.001 |

**Abbreviations:** CIHR=the cumulative incidence rate of hematological recurrence; CIR=the cumulative incidence rate of relapse including hematological and extramedullary; LFS=leukemia-free survival; OS=overall survival; New risk scoring system was the number of risk factors, including T-ALL, CR2 or above stage at HSCT, pre-HSCT MRD positive, and pre-HSCT CNS involvement and divided into four distinctive risk groups: low-risk group 0, intermediate-risk group 1, high-risk group 2 and extremely high-risk group 3 to 4; HR=hazard ratio; CI=confidence interval;

* All variables were first included in the univariate analysis; only variables with *P*< 0.1 were included in the Cox proportional hazards model with time-dependent variables.

**Table S3. Effects of CNS involvement pre-HSCT on prognosis in each subgroup**

| subgroup | CIHR | | CIR | | LFS | | OS | |
| --- | --- | --- | --- | --- | --- | --- | --- | --- |
|  | 1. year rate   (95%*CI*) (%) | P value | 5-year rate  (95%*CI*) (%) | P value | 5-year rate (95%*CI*) (%) | P value | 1. year rate   (95%*CI*) (%) | P value |
| B-ALL |  | 0.006 |  | 0.001 |  | 0.002 |  | 0.067 |
| Negative CNS pre-HSCT | 21.5(19.0-24.0) |  | 22.5(19.7-25.3) |  | 61.9(59.0-64.8) |  | 67.6(64.9-70.3) |  |
| CNS involvement pre-HSCT | 36.8(24.1-49.5) |  | 40.4(27.5-53.3) |  | 43.9(31.0-56.8) |  | 56.1(43.2-69.0) |  |
| T-ALL |  | <0.001 |  | <0.001 |  | 0.001 |  | <0.001 |
| Negative CNS pre-HSCT | 25.4(20.2-30.6) |  | 26.9(21.7-32.1) |  | 56.8(50.9-62.7) |  | 59.3(53.4-65.2) |  |
| CNS involvement pre-HSCT | 64.7(88.9-40.5) |  | 64.7(40.5-88.9) |  | 23.5(3.3-43.7) |  | 23.5(3.3-43.7) |  |
| CR1 |  | 0.005 |  | <0.001 |  | 0.001 |  | 0.025 |
| Negative CNS pre-HSCT | 19.7(17.7-21.7) |  | 20.9(18.9-22.9) |  | 63.9(61.2-66.6) |  | 68.7(66.0-71.4) |  |
| CNS involvement pre-HSCT | 36.2(22.6-50.0) |  | 40.1(26.2-54.0) |  | 43.9(30.2-57.6) |  | 53.9(40.0-67.8) |  |
| CR2 and above |  | 0.045 |  | 0.046 |  | 0.192 |  | 0.319 |
| Negative CNS pre-HSCT | 39.5(31.9-47.1) |  | 39.8(32.5-47.1) |  | 40.4(33.0-47.8) |  | 47.0(39.4-54.6) |  |
| CNS involvement pre-HSCT | 58.3(37.7-78.9) |  | 58.3(37.7-78.9) |  | 29.2(11.0-47.4) |  | 37.5(18.1-56.9) |  |
| Pre-HSCT MRD negative |  | 0.003 |  | 0.001 |  | 0.001 |  | 0.010 |
| Negative CNS pre-HSCT | 19.1(16.3-21.9) |  | 19.7(16.9-22.5) |  | 64.4(61.5-67.3) |  | 68.5(65.6-71.4) |  |
| CNS involvement pre-HSCT | 34.5(22.1-46.9) |  | 36.2(23.6-48.8) |  | 46.6(33.9-59.3) |  | 53.4(40.5-66.3) |  |
| Pre-HSCT MRD positive |  | <0.001 |  | <0.001 |  | 0.003 |  | 0.036 |
| Negative CNS pre-HSCT | 33.7(28.2-39.2) |  | 36.4(30.9-41.9) |  | 48.1(42.2-54.0) |  | 56.8(51.1-62.5) |  |
| CNS involvement pre-HSCT | 75.0(51.8-98.2) |  | 81.3(59.8-100.0) |  | 12.5(0-28.8) |  | 31.3(8.6-54.0) |  |

**Abbreviations:** CIHR=the cumulative incidence rate of hematological recurrence; CIR= the cumulative incidence rate of relapse including hematological and extramedullary; OS=overall survival; LFS=leukemia-free survival, HSCT=hematopoietic stem cell transplantation; ALL=acute lymphoblastic leukemia; CNS involvement= blasts from CSF were detected; negative CNS pre-HSCT= blasts from CSF were not detected before transplant; HR=hazard ratio; CI=confidence interval;

* All variables were first included in the univariate analysis; only variables with *P*< 0.1 were included in the Cox proportional hazards model with time-dependent variables.
